# Widening or narrowing inequalities? The equity implications of digital tools to support COVID‐19 contact tracing: A qualitative study

**DOI:** 10.1111/hex.13593

**Published:** 2022-09-05

**Authors:** Catherine A. O'Donnell, Sara Macdonald, Susan Browne, Alessio Albanese, David Blane, Tracy Ibbotson, Lynn Laidlaw, David Heaney, David J. Lowe

**Affiliations:** ^1^ General Practice and Primary Care, School of Health and Wellbeing University of Glasgow Glasgow Scotland; ^2^ Public and Patient Involvement and Engagement Group, College of Medicine, Veterinary and Life Sciences University of Glasgow Glasgow Scotland; ^3^ Rossall Research and Consultancy Ullapool Scotland; ^4^ NHS Greater Glasgow and Clyde, Digital Health and Care Institute University of Glasgow Glasgow Scotland

**Keywords:** contact tracing, COVID‐19, digital tools, inequalities, marginalized populations

## Abstract

**Background:**

As digital tools are increasingly used to support COVID‐19 contact tracing, the equity implications must be considered. As part of a study to understand the public's views of digital contact tracing tools developed for the national ‘Test and Protect’ programme in Scotland, we aimed to explore the views of groups often excluded from such discussions. This paper reports on their views about the potential for contact tracing to exacerbate inequalities.

**Methods:**

A qualitative study was carried out; interviews were conducted with key informants from organizations supporting people in marginalized situations, followed by interviews and focus groups with people recruited from these groups. Participants included, or represented, minority ethnic groups, asylum seekers and refugees and those experiencing multiple disadvantage including severe and enduring poverty.

**Results:**

A total of 42 people participated: 13 key informants and 29 members of the public. While public participants were supportive of contact tracing, key informants raised concerns. Both sets of participants spoke about how contact tracing, and its associated digital tools, might increase inequalities. Barriers included finances (inability to afford smartphones or the data to ensure access to the internet); language (digital tools were available only in English and required a degree of literacy, even for English speakers); and trust (many marginalized groups distrusted statutory organizations and there were concerns that data may be passed to other organizations). One strength was that NHS Scotland, the data guardian, is seen as a generally trustworthy organization. Poverty was recognized as a barrier to people's ability to self‐isolate. Some participants were concerned about giving contact details of individuals who might struggle to self‐isolate for financial reasons.

**Conclusions:**

The impact of contact tracing and associated digital tools on marginalized populations needs careful monitoring. This should include the contact tracing process and the ability of people to self‐isolate. Regular clear messaging from trusted groups and community members could help maintain trust and participation in the programme.

**Patient and Public Contribution:**

Our patient and public involvement coapplicant, L. L., was involved in all aspects of the study including coauthorship. Interim results were presented to our local Public and Patient Involvement and Engagement Group, who commented on interpretation and made suggestions about further recruitment.

## 1 BACKGROUND

The COVID‐19 pandemic has deepened pre‐existing health inequalities.^1^, ^2^, ^3^ The risk of contracting COVID‐19 and experiencing worse outcomes, such as hospitalization, intensive care unit admission and mortality, is greater for those living in poverty and for minority ethnic groups.^3^, ^4^, ^5^ Essential workers, including lower‐paid occupations, and those living in overcrowded housing are at greater risk.^6^, ^7^ Mitigations, in particular, social distancing and working from home, and the ability to self‐isolate are all more difficult for marginalized populations, with intersections between poverty, multiple disadvantage and ethnicity.^2^, ^8^, ^9^, ^10^ A key strategy for population control is testing and contact tracing to break chains of transmission, but this also has the potential to exacerbate inequalities. In England, reporting of positive cases and contact tracing was lower in areas of deprivation.^11^ Nevertheless, there has been little consideration of the inequities that might occur with contact tracing interventions. A rapid review of contact tracing interventions for a range of infectious diseases (including COVID‐19) found that no intervention considered the potential for increasing health inequities and there were no attempts to mitigate potential inequities in their design.^12^ Others have also commented on the potential for digital technologies such as proximity apps to disadvantage certain groups.^13^ This digital divide is well recognized, with certain groups including minority ethnic populations, older people and those on low incomes all likely to be disadvantaged by technological approaches to health and health care.^14^ It is this potential for contact tracing and its associated digital interventions to exacerbate inequalities that is the focus of this paper.

Contact tracing is a mainstay of public health surveillance.^15^ Following the identification of a positive case, contact tracing seeks to rapidly identify all their contacts within a predefined period; those identified are tested and/or self‐isolate.^16^ Contact tracing has been used in the surveillance of many communicable diseases.^17^, ^18^ However, its history is problematic, with different responses to different population groups, breaches of trust in data use and confidentiality and lack of privacy for contacts.^15^, ^18^

The COVID‐19 pandemic presents particular challenges for contact tracing. Cases may be asymptomatic, population spread can be rapid and the prevalence of infection high. Contact tracing has to occur at speed and scale to cut transmission chains.^16^, ^19^ Previously, contact tracing relied on manual approaches, with healthcare professionals or others collecting information directly from individuals. More recently, digital approaches have been developed and used in outbreaks of Ebola, tuberculosis and in sexual health services.^17^, ^20^, ^21^, ^22^ COVID‐19 has accelerated this shift towards digital tools to enable large‐scale contact tracing.^23^, ^24^, ^25^

Attention has focused on the development and use of COVID‐19 contact tracing proximity apps.^26^ Downloaded onto smartphones, these use Bluetooth to identify when another phone has the app active in close proximity. Close proximity is determined by distance (generally ≤2 m) and contact time (usually ≥15 min), to rule out passing contacts, for example in a shop or in the street. If a user then tests positive for COVID‐19 and uploads that information onto the app, close contacts are automatically notified. There are, however, challenges to their use. First, a substantial proportion of the population has to download and use the app—in the United Kingdom, estimated to be about 80% of smartphone users (37 million people).^19^ Second is storage of contact data. Decentralized approaches retain a record of device interactions on the smartphone itself; centralized systems hold data on cloud servers owned by commercial companies (commonly Google or Apple), raising concerns about data privacy and confidentiality.^19^ Third, there are concerns about increased digital inequality in terms of access and use of apps.^13^

Studies have explored public views of contact tracing apps. While many recognized that, in a pandemic, such apps may be necessary to control infection, there were reservations about data storage, privacy and ethics.^27^, ^28^, ^29^, ^30^, ^31^ A longitudinal survey of 405 Irish citizens before and after the launch of a contact tracing app found that citizens' willingness to download the app was shaped by their beliefs about health benefits and social good. Privacy concerns reduced their willingness to use the app.^32^ A cross‐sectional survey of users of the NHSX app in England found that while 62% felt that it met their expectations, 25% had not opened the app after downloading it. Concerns included the privacy of the information required and some wanted more information on data storage and sharing.^33^ Similar results were reported by Dowthwaite et al.^34^ Of 1000 study participants, 50% downloaded the NHSX app onto their phone; however, 36% had either not downloaded it or downloaded it and then deleted it. More respondents from minority ethnic populations had deleted the app and more older people had not downloaded it.

Qualitative studies have explored people's concerns in more detail. Over 300 qualitative interviews conducted across nine European countries in April and May 2020 identified a spectrum of views regarding COVID‐19 contact tracing apps, ranging from opposition, through scepticism about their feasibility, to support.^35^, ^36^ Similar views were reported in focus groups held in May 2020 with UK adults. Those concerned about privacy, uptake of the app and stigma were most likely to say that they would not download a contact tracing app.^37^

While this study offers important insights, we believe that there are limitations especially in relation to the study population. Some studies were conducted before such apps were available, leaving the public to think about their use hypothetically. While study populations were broadly representative in terms of gender and age, they were less likely to consider ethnicity and, where information was given, participants tended to be well educated, indicating more socioeconomically affluent populations. These are all important considerations when trying to develop approaches that will not further exacerbate COVID‐19 health inequalities.^38^

### 1.1 Digital contact tracing NHS Scotland

In the summer of 2020, NHS Scotland developed a proximity app for contact tracing.^39^ Developed in collaboration with the Digital Health & Care Innovation Centre (DHCI), this followed the model developed in Ireland. Proximity alerts were the main function and there was no requirement to enter personal data.^39^, ^40^, ^41^ Data storage was decentralized, with data held on the mobile phone for 14 days. Called ‘Protect Scotland’, the app was launched in September 2020 as part of NHS Scotland's ‘Test and Protect’ programme.

A suite of digital tools was also commissioned to enhance contact tracing. This included a web‐based interface to allow people informed of a positive test result to report information on their recent contacts (https://www.dhi-scotland.com/projects/covid-19-test-and-protect-tap/). This information was accessible to contact tracers and used in the subsequent contact tracing call. All aspects of the contact tracing service are part of NHS Scotland. As part of the development of the Test and Protect programme and its digital tools, we were commissioned by the DHCI to seek the views of a wide range of people, with a focus on those from marginalized, multiply disadvantaged and/or minority ethnic communities. Our aim was to seek their views with respect to the Test & Protect programme and the digital contact tracing reporting tool, whether they would use it, any concerns they may have and what they thought of the prototypes to inform its development. During this study, DHCI asked us to look at particular parts of their programme, for example, the readability of the information presented and the privacy statement, but had no role or influence on the conduct of the research or the analysis and interpretation of the data. During data collection, people spoke at length about the potential for contact tracing to exacerbate inequalities. These are the findings that we present in this paper.

## 2 METHODS

This was a qualitative study involving semi‐structured interviews and two focus groups with members of the public, and semi‐structured interviews with key informants representing community organizations. All participants were aged over 18 years. Data collection occurred in June and July 2020, while the proximity app and contact tracing tools were in development.

### 2.1 Recruitment

We began with key informant interviews. The research team used their links and knowledge of community‐based organizations working with marginalized and/or multiply disadvantaged communities to create a purposive sample of informants. Those who agreed to participate worked with minority ethnic groups; asylum seekers and refugees; survivors of domestic abuse; addiction services; homeless services; people with long‐term conditions; children and young people; and people experiencing multiple disadvantage, including extreme poverty. We also interviewed a rural GP and a GP working in an area of socioeconomic deprivation. All approached agreed to be interviewed or, if unavailable, suggested someone else in the organization.

Key informants then facilitated access to people in the communities that they worked with. The research team also used their links with organizations and people to recruit members of the public. Those interested contacted the research team for more information and, if they agreed to participate, a suitable time for interview was arranged. Focus group recruitment was facilitated through organizational links, one working with minority ethnic communities and the other working with people experiencing multiple disadvantage.

### 2.2 Data collection

Interviews and focus groups were conducted via Zoom at a time suitable to participants; most participants were interviewed in their own home. Topic guides and information materials were developed in advance by the research team, including our patient and public involvement (PPI) coapplicant L. L., drawing on available literature at the time but also including questions and topics suggested by the developers of the digital tools, to assist them in developing the tools. While the topic guide was not piloted before use, we discussed it throughout data collection and added prompts where necessary, for example, in relation to data privacy. During the interviews and focus groups, we shared screenshots of the digital tool with participants. For example, we shared and discussed privacy statements to determine their clarity and understandability and showed participants a set of screenshots with the information and layout that would be contained in the app. Interim results were shared with our Patient and Public Involvement and Engagement group for comment, who made suggestions on data interpretation and on future recruitment strategies.

Participant information sheets and consent forms were emailed to those wishing to participate before data collection. Completed consent forms were emailed back to the research team; however, verbal consent was also recorded at the beginning of each interview or focus group. Interviews and one focus group were conducted by S. B. and A. A., with one focus group and two interviews conducted by C. O'D. The interviews and focus groups were recorded, with consent, and downloaded to the University of Glasgow server for secure storage. Members of the public received a £20 shopping voucher as a token of our thanks for their time. Interviews lasted between 40 and 60 min; focus groups lasted approximately 90 min.

### 2.3 Data analysis

Analysis utilized the framework approach to enable rapid feedback to the app developers and to facilitate comparison across participants.^42^, ^43^ A lack of resource meant that the interviews and focus groups were not transcribed; analysis instead used the sound recordings as the primary source of data.^44^, ^45^, ^46^ Using the framework approach and regular coding discussions within the team ensured that rigour and transparency were maintained during the coding process. The recordings were also augmented by fieldnotes made at the time by those conducting data collection.^47^

Analysis followed the five steps of the framework approach: First, we became familiar with the data by listening repeatedly to the recordings, both those who collected the data (authors C. O'D., S. B. and A. A.) and other members of the team. A thematic framework was developed, drawing on the areas covered by the topic guide but also issues that arose during the interviews (e.g., in relation to information sharing). These formed a set of eight charts in Excel. Each broad theme was broken down into subthemes. The thematic framework was then applied to each interview and focus group, with data extracted into the Excel sheets, along with researchers' interpretation of the data. Extracted data included a timestamp to allow researchers to identify the data in the sound recording, to facilitate data checking and extraction of quotes. These became our main data source for report and paper writing. As our particular interest was the potential for contact tracing to exacerbate inequalities, we used the PROGRESS framework as a lens to inform the interpretation of our findings.^48^ The PROGRESS framework was developed to ensure that equity was explicitly considered in the design of new intervention and programmes by highlighting factors that impact on health opportunities and outcomes and that can exacerbate inequalities: place of residence, race/ethnicity/culture/language, occupation, gender/sex, religion, education, socioeconomic status and social capital. The PROGRESS framework helped us consider (i) what groups or characteristics or (ii) social or geographical variables were discussed and how these impacted on people's ability to engage with contact tracing and digital tools. For example, were some issues more pertinent to younger or older people? Were there different experiences as a result of gender, ethnicity or socioeconomic status?

Findings were shared across the research team, with consequent discussion of interpretation. Our PPI coapplicant offered insights into the findings and our interpretation, for example, by suggesting that we pay more attention to the practical implications of self‐isolation, including affordability. To protect anonymity, all participants are referred to by a letter (K for key informant; P for public), and for public participants, gender and broad age category.

## 3 RESULTS

### 3.1 Respondents

We recruited 42 participants: 13 key informants and 29 members of the public (16 interviews and 13 in each focus group). We aimed for diversity amongst public participants, although women predominated and we had few aged under 30 years (Table 1). Analysis identified five themes: views and knowledge of contact tracing; language and digital access; data governance and privacy; inequalities related to contact tracing; and barriers to self‐isolation.

**Table 1:** Characteristics of public participants (*n* = 29)

Gender	
Female	21
Male	8
Age group	
Younger (<30 years)	3
Middle‐aged (30–60 years)	18
Older (>60 years)	8
Ethnicity	
White Scottish	20
South Asian/Scottish South Asian	6
Other ethnicity	3
Disability or long‐term condition	
Yes	5
No	24
Location	
Urban/suburban	24
Semi‐rural or rural	5
Employment	
Working	15
Retired	5
Unemployed	7
Student	2

### 3.2 Views and knowledge of contact tracing

Public participants generally viewed contact tracing and proximity apps as a good idea, even if they were not clear about the process. There was no one who said it was not a good idea. Some framed use as a responsibility to their family, friends and wider community, and others as a ‘civic duty’ (P2, older male).

Key informants had more concerns. One worked with a charity supporting women experiencing domestic abuse and raised concerns about the risks to women's personal safety. First, women may not want their perpetrator to know that they had been in contact with others. Second, women may not want to disclose a recent contact with their perpetrator, especially if there was a court order forbidding contact with that person, for fear of repercussions.

They may not want to tell you honestly because the perpetrator may be threatening [them]. (K1)

A second issue was trust. Several key informants stated that the communities that they worked with were already marginalized and often distrusted official organizations, so would be less likely to engage with contact tracing. Concerns included confidentiality of the data that they had to provide, how that would be used and ramifications for their contacts.

…it's really complicated. First of all, there is a huge issue about trust. So, many of the people we work with have really struggled over the lifecourse with people in positions of power… Dominant discourses are around this idea of ‘We are a society, we've got to work together to fight this’. Many of the people that I work with have learned to distrust that. (K5)

A public participant who volunteered with asylum seekers also described the lack of trust that this population often has with official organizations. Thus, the negative impact of previous poor experiences with official organizations needs to be acknowledged when trying to promote contact tracing across population groups.

…people under immigration rules and control might find questions around where they have been quite [pauses]… a bit worrying and that might force them not to want to disclose [information] for their own safety. (P22, middle‐aged woman)

### 3.3 Language and digital access

Using these tools requires both language and digital access skills. Missing one or both of these, it was suggested, would widen inequalities. For example, although information on the Test and Protect programme was available in several languages, the web‐based interface for contact information was only in English, disadvantaging those whose first language was not English. There were also concerns that some of the information required a reasonable degree of literacy.

So language wise, there needs to be more than just English or its going to miss an entire swathe of the population… So, they will definitely need it in other languages. On the literacy, this is why I was saying it's too wordy, uses too many big words that people don't routinely use in their everyday language…. you have to assume, you're going to be looking at at least 25% [of users] with low literacy. (K3)

As interviews were held via Zoom, our participants had good digital literacy. Almost all the public participants had a smartphone, all regularly used the internet and most expressed confidence in using online platforms. Most had no issues using digital tools or websites on their smartphone, tablets or laptops. One younger participant commented that under 30s ‘spend their lives on‐line’ (P12, younger male). An older participant commented that the pandemic had driven even older people to develop their digital literacy skills.

I think for us, for my generation ‐ I'm obviously older ‐ this has all been terribly new and we've had to learn very, very quickly. Emm, they are trying to get me onto Microsoft Teams, we're having a terrible problem. But it turns out may be the system, rather than me ‐ you understand? I've got onto Zoom no problem. But these are all things that a couple of months ago, I wouldn't have had a clue how to use. (P9, older woman)

They did, however, contrast their relatively good experience with those who did not have such ready access. Participants living in rural areas were concerned about internet access and connectivity. All recognized different barriers to digital literacy, some of which intersected. Several commented that more elderly people might struggle with the digital elements of the Test and Protect programme. Those taking part in the South Asian focus group were particularly concerned about the lack of digital capability amongst older members of their community, who often only communicated by telephone. However, some key informants cautioned against thinking that only older people had difficulties accessing the internet.

The older generation in our community … some of them just know how to make or receive a call… some of them don't know how to use the internet or anything. How are we going to get to those people and how do we get the information there? Some of them will be living alone. So if somebody is living with their family, they can help them. But if someone is living on their own, how do we get them? (P21, younger female)

[Name of charity] have become very aware that there's a large percentage of people who don't have access to online, and even if they do, they're not very sure how to use it. The assumption is [name of charity] works with older people, it's not true, and it's not just older people that don't have access to online. (KI4)

An important barrier to digital access was poverty, affecting all age groups. Key informants representing organizations working with poorer communities reiterated that smartphone ownership did not guarantee equality in digital access. Having the financial resource necessary for unlimited data was thought unlikely for those living in severe socioeconomic deprivation. Many people used pay‐as‐you go contracts and could have periods of time where they could not afford to pay for mobile phone top‐ups. Phone sharing was also raised as a barrier.

…digital exclusion is a reality and takes a number of forms. One is people don't have any access to digital equipment at all and they don't do business by email. I think in terms of the various tracing and symptom apps that are around, my impression is that they are not really on the radar of a lot of folk in lower socioeconomic groups. They are more of a phenomenon amongst a mobilised middle‐class. (K2)

Interlinked with the issues of digital access and affordability was trust in how data would be collected, stored and used and trust in the organizations involved. This is discussed next.

### 3.4 Data governance and privacy

Public participants were, in general, trusting of the contact tracing process and the digital tools to support that process. They were not unduly concerned at submitting their personal data to a digital platform, especially as that required a unique code sent to them by email or SMS text message from Test and Protect at the same time as their positive test result. Knowing that the Test and Protect programme and its digital tools were part of NHS Scotland also promoted trust.

Public participants were reassured by the privacy statement on the app, describing it as ‘a fairly standard statement' (P3, older male). Most were also reassured by the ability to click onto a longer privacy statement held on the Scottish Government's information governance site, if they so wished.

It's making a promise to me that my data will be securely held under the data protection legislation, which is what I would expect to see. And that you are only going to use my data for the reason that you have given me and under GDPR regs; that's about all I need to know. (P5, older female)

However, several participants commented on the need to be clear about how long their data were stored for and who could access the data. They wanted reassurance that their data would not be shared with third parties external to the NHS. Some felt that this should be explicit in the confidentiality statement on the contact tracing site, which, as one participant commented:

[it] should maybe be expanded to say ‘and this information will not be used outside the NHS and not given to a third party’…. A lot of people will have notions in their head about things [data] being sold. (P1, middle‐aged female)

Key informants, including those working with children and young people, with minority ethnic communities, with asylum seekers or those with addiction problems, said that the people they represented would not read such a long privacy statement. Again, though, it was clear that people would want a clear, straightforward indication of who could access their data, for how long and how it would be used. Groups experiencing multiple disadvantage, such as victims of domestic abuse, required particular reassurance about how their data were being used and who had access to it.

It goes back to the communication, and the buy‐in from the people…. People want safety, they want to be OK. As long as they seek reassurance around the data, and what it's used for, and how long and where it's [the data] going…. They would be appreciative of that. (K11)

Key informants were also clear that trust in the system, not just the app, was essential, as many people feared that their data might be passed on to carry out benefits checks or, for those seeking asylum, to the UK Home Office. One informant suggested that people living with addictions would be concerned that disclosures around encounters involving drug use could affect access to their children. Thus, many groups require reassurance about the safety and confidentiality of their data, although for different reasons.

### 3.5 Inequalities related to contact tracing

Participants talked about the likely dilemmas faced by having to give information about recent contacts, especially if those contacts were in a potentially marginalized or vulnerable situation. First, recent contacts were likely to be friends or family. Second was the knowledge that some contacts might have arisen though activities that (at the time of data collection) were not permitted, for example, larger household gatherings. Finally, an individual's contacts might not want their personal information given to Test and Protect. Some suggested that they would deal with this by telling their close contacts that they were being tested and, if positive, would share contact information.

I don't have qualms about giving my own data but I feel a wee bit uncomfortable about giving someone else's. …I'd want to do them the courtesy of letting them know. (P4, middle‐aged female)

This raised an additional dilemma—what to do if friends or family did not want their details given to Test and Protect? While some said that they would still pass on contact details because ‘we all have a part to play’ (P8, middle‐aged female), others were less sure and acknowledged that some disclosures may be difficult. It was also acknowledged that some people may find it difficult to self‐isolate, due to the nature of their job and the potential to be unpaid during their isolation.

Depends on the situation of the individual person and maybe their contact with other people, sometimes they just want to keep it secret… you never know what people are doing in their life, so sometimes they may not want to disclose what they've been up to or who they've been meeting. There will be a privacy issue for some people. (P20, middle‐aged female)

Although contact tracers do not say who has passed on contact details, there was also a fear that this would happen informally within social networks or because the population size was small, for example, in rural areas. A few participants, especially amongst key informants, discussed the possible stigma of testing positive.

The age group 45 and above will be much less likely to share contacts. Stigma in these [minority ethnic] communities will play a big role. (K9)

Thus, inequalities could be perpetuated in different ways – through socioeconomic position, ethnicity/race or through geography.

### 3.6 Barriers to self‐isolation

At the time of data collection, Scottish Government advice was to isolate for up to 14 days if one had COVID‐19 or were a close contact of a positive case. It was recognized that there were barriers to self‐isolation and these affected people differently according to their circumstances. While most public participants interviewed felt that they had strong support networks that could assist them if they required groceries and essential supplies, there was recognition that others may not have such support. One participant commented that successful isolation required reliance on local communities for help and talked about being reticent to ask strangers for help. The impact of self‐isolation on people's mental health and well‐being was also raised, particularly if they were asked to self‐isolate more than once. Self‐isolation was also recognized to have financial implications including loss of earnings and a lack of sick pay, particularly for people on zero hours contracts and precarious employment.

If people feel as though they are not going to get financial support, and benefits, and if they have to stop work and stay at home then folk are not going to be honest about it and they are not going to co‐operate, are they? I have heard some horror stories about the time, it taking a long time for benefits to come through or grants to come through if people are self‐employed and running small businesses. (P6, older female)

One participant talked about his own experience when he had to self‐isolate after contracting COVID‐19 and reflected on whether this was sustainable if people were asked to self‐isolate multiple times.

For me, personally, I don't think it would mean a lot because I am paying digs [rent] at the moment but my Mum could always lessen them depending on how much my wages are. But I obviously can imagine that for people supporting a family and having more responsibilities than me, that could be devastating to take two weeks off. Cos I think I lost £200‐£300 in the week I was off. (P12, younger male)

Other, practical barriers to self‐isolation were discussed that would make it harder for those living in poverty or experiencing multiple disadvantage to self‐isolate. These included the practicalities of following official advice to get food deliveries and the difficulties of maintaining self‐isolation when living in overcrowded housing.

Well, there's a charge for food to be delivered. There's also a minimum spend on food to be delivered which people living in poverty never reach. They tend to shop frequently in small amounts because different benefits and different payments, and even employment when its insecure and on a zero hours basis, can come through at different times. …Local shops don't offer delivery and local shops are where people tend to shop. So again, that's a requirement that middle‐class people can easily adhere to, people living in poverty would struggle to adhere to. (K3)

The issues are the practical side of it. You are talking about isolation for example, how do people isolate when they are in maybe overcrowded housing and, emm and also they have children …. That would make it difficult for people. (K11)

## 4 DISCUSSION

### 4.1 Summary of findings

We found strong support amongst participants for contact tracing for COVID‐19 and for the use of self‐report digital tools to facilitate that process. Applying an equity lens to our findings highlighted the clear potential for widening and deepening existing inequalities.^3^ The PROGRESS framework highlighted place of residence, race/ethnicity/culture/language, occupation, gender/sex and socioeconomic status as limiting people's ability to participate fully in contact tracing, access the digital tools designed to support Test and Protect and adhere to self‐isolation if required.

Our findings identified population groups who could be particularly challenged by contact tracing, including victims of domestic violence, minority ethnic populations, asylum seekers and refugees, those working on zero hours contracts and those experiencing multiple disadvantage. These intersect, with poverty playing an overarching role in the ability of individuals and communities to respond to contact tracing. Poverty impacted on people's ability to afford mobile phones and the data required to access the digital tools. This was not restricted by age. Poverty also affected people's ability to self‐isolate if required due to a lack of money to access support and the lack of financial resilience to lose wages. Concerns were expressed about identifying contacts who were self‐employed or on zero hours contracts, who may struggle to self‐isolate. Language and literacy barriers also affected many groups, either because English was not a first language or because people did not have the necessary literacy levels to read and understand the digital information provided. Although findings from this study were used to modify the language used in the digital tools, for example, modifying some of the description in relation to COVID‐19 symptoms, this suggests that more work is required to ensure that contact tracing information and digital tools are accessible.

Trust in the organizations in charge of Test and Protect and in how data were being collected and used was key. Many groups were generally distrustful of statutory organizations because of previous experience distrustful about use of their data. This view was more prevalent amongst the key stakeholders than with members of the public, perhaps reflecting the population groups that the stakeholders worked with. Public trust in statutory organizations, including the NHS and government, is often fragile and depends on previous experience.^49^ Trust, however, is essential in adopting the measures designed to control the spread of COVID‐19.^50^ We, therefore, suggest that targeted information campaigns using trusted members of communities identified as marginalized should be implemented to give people the opportunity to ask questions, raise concerns and—crucially—have those concerns addressed.

### 4.2 Strengths and limitations

A strength of this study was our ability to involve public participants and key stakeholders who were from, or represented, a diverse group of people who are often at the margins of society, including those experiencing severe and multiple disadvantage, homelessness or those from minority ethnic communities. Applying an equity lens using the PROGRESS framework identified the clear potential for widening and deepening the inequalities that are already present in our society and that COVID‐19 has exacerbated.^3^

Limitations included that we recruited key stakeholders rather than people with lived experience of some situations, for example, living with domestic violence. As interviews and focus groups were all conducted by Zoom, our sample had at least a degree of digital literacy. While participants reflected on the likely impact of having poorer digital literacy or lack of access, they could not fully represent the views of people in that situation. Lack of formal transcription was another limitation and could result in more superficial coding and analysis; however, we used recognized methods to approach analysis and interpretation of findings.^44^, ^45^, ^46^ In addition, the use of the PROGRESS framework aided interpretation of our data. Finally, given the timeframe of data collection, we did not include anyone who had actual experience of using Test and Protect and its associated digital tools, which could identify other barriers, and facilitators, to contact tracing that we did not uncover.

### 4.3 Comparison with previous literature

Previous work on the development of contact tracing tools for COVID‐19 focused on public views of proximity tracing apps, often before such apps were fully developed.^27^, ^28^, ^29^, ^30^, ^31^ However, few studies included people from marginalized populations. Our work is the first to actively recruit people, or their representatives, from a range of marginalized groups. While the digital tools had not yet been launched for public use, we were able to share the prototypes with participants, so they were able to see—and reflect on—the tools that would be implemented. Our work shows that the inequalities associated with COVID‐19 infections and adverse health outcomes—namely, poverty, multiple disadvantage and ethnicity—also play a role in people's ability to participate in contact tracing^1^, ^2^, ^3^ and mirror well‐known barriers to accessing and using digital technology more generally.^13^, ^14^ A consequence of contact tracing, if positive or in contact with a positive case, is self‐isolation. The findings reported here indicate the difficulties that poverty and precarious employment place on people, to the extent that some would be reluctant to disclose either their status or that of others. Financial hardship and lower socioeconomic status were two of the factors associated with rates of nonadherence in contact sharing across the United Kingdom.^51^ Several studies reported on the public's concerns about data privacy and whether data would be shared with other organizations.^32^, ^33^, ^34^ Participants in our study appeared to be reassured about data privacy, as the data are held under the governance framework of NHS Scotland, which is generally regarded as a trustworthy organization.

This study demonstrates that contact tracing has ethical and moral dimensions that people need to navigate. Some of this has been articulated. including whether digital approaches to contact tracing exacerbate discrimination and inequalities and whether disclosing contacts can result in stigma or infringe people's privacy.^52^, ^53^ Gasser et al.^25^ have mapped the legal and ethical considerations that they recommend the developers of COVID‐19 digital health technologies should consider. These include ensuring public benefit, protecting privacy, avoiding discrimination and preventing digital inequality. Our findings demonstrate that preventing inequality needs to considered across the spectrum of contact tracing, including self‐isolation.

Since this study was conducted, there have been changes to the way in which the COVID‐19 pandemic is handled in the United Kingdom, including in Scotland. The Test and Protect programme, and contact tracing, has recently been suspended. However, it is always possible that the emergence of a new, more potent variant of COVID‐19 will require contact tracing to be reinstated. Contact tracing also remains a key public health strategy for other communicable diseases. We believe that our findings will have salience for other, future programmes where those experiencing disadvantage and marginalization may struggle to engage.

## 5 CONCLUSIONS

The impact of contact tracing and its associated digital tools on marginalized populations requires monitoring. This should include not only contact tracing itself but also the ability of people to self‐isolate. Support for self‐isolation is a vital part of contact tracing. Without that, people will make difficult decisions not to participate or may withhold information on contacts. Our work showed that it was possible to seek the views and opinions for those in marginalized communities and use the information to improve the design of the digital tools developed; this participatory approach allows the system to be ‘sense‐checked’ with as diverse a group of people as possible. Regular clear messaging political leaders and from trusted members of communities who are marginalized about contact tracing could help maintain trust and participation in the programme. Finally, our findings can be used to inform the development of future programmes of contact tracing for other conditions, to ensure that public health responses are both scalable and meet the needs of all population.

## AUTHOR CONTRIBUTIONS

Catherine A. O'Donnell, Sara Macdonald, David Blane and David J. Lowe conceived the study and wrote the protocol. Catherine A. O'Donnell obtained ethical approval. Susan Browne and Alessio Albanese led data collection, with support from Catherine A. O'Donnell, David Blane and David Heaney. Catherine A. O'Donnell, Susan Browne and Alessio Albanese led the analysis; Catherine A. O'Donnell and Sara Macdonald drafted the paper. Tracy Ibbotson and Lynn Laidlaw ensured that the work was guided by patient and public experience. All authors contributed to the interpretation of data and revision of the paper. Lynn Laidlaw provided key insights as the patient and public involvement representative.

## CONFLICT OF INTEREST

The authors declare no conflict of interest.

## ETHICS STATEMENT

The study was approved by the Research Ethics Committee, College of MVLS, University of Glasgow, project number 200190168.

## Data Availability

Data are available upon reasonable request to Kate.O'Donnell@glasgow.ac.uk. Original sound files cannot be shared as they contain identifiable material.
